# The INDUS knee prosthesis: Prospective multicentric trial of posteriorly stabilized high-flex design: Two years follow-up

**DOI:** 10.4103/0019-5413.73666

**Published:** 2011

**Authors:** Ashish Anand, A Ravi Raj

**Affiliations:** *Joint Replacement and Sports Medicine Unit, Fortis Hospitals, Delhi, India*

Sir,

We read the article “The INDUS knee prosthesis: prospective multicentric trial of a posteriorly stabilized high-flex design: Two years follow-up” by Sancheti KH *et al*.^1^ with interest. We compliment the authors for a well written prospective study. We have a few concerns regarding the prosthesis design and the clinical outcome described by them for Indian patients.

It is not uncommon to see patients with very advanced osteoarthritis with gross, neglected deformities, with very limited pre-operative range of motion, confined to wheelchair or bed for many years in this part of the world. The patients presenting even in late stages anticipate sitting crosslegged and squatting, in the postoperative period. The authors’ series does not describe such severe deformities. The authors have discussed the preoperative deformity as ranging from 32 degrees of varus to 18 degrees of valgus (the femorotibial angle). It is not clear whether the authors have excluded severe deformities and patients with restricted pre operative range of motion and obese patients. This leaves the reader with the dilemma of choosing such a high flex design, which theoretically promises the benefits of squatting and sitting cross legged.

Authors in their study have described the design modifications of the prosthesis to achieve a mean flexion of 135 degrees without compromising the stability, which allows the patients activities such as squatting and sitting cross legged. However the authors in their two years follow-up have described 24 knees in their series, having a flexion of less than 100 degrees. The cause of such a flexion loss at early follow-up has not been discussed in their work.^1^ Did these patients have restricted movements in the pre operative period? This leaves the reader wondering why there was flexion loss in these patients despite the use of a highflex design. The significance lies in the fact that if there is restricted range of motion preoperatively, is there any advantage of such a high-flex design? Many studies have shown the clinical and functional outcome of a fixed and mobile bearing total knee arthroplasty to be similar.^2^–^4^ Studies also indicate that the preoperative functional status is an important indicator in the post operative outcome and function in patients undergoing total knee arthroplasty.^5^,^6^ It is not clear in the manuscript whether the design of the prosthesis *per se*, can increase the postoperative function and range of motion in a knee that had restricted range of motion and function in the pre operative period?

The INDUS knee prosthesis described does not have an option of using extenders with the femoral component. The study includes 44 patients with rheumatoid arthritis (RA) and the authors have not described any of these patients as having poor bone quality, a common finding in such patients. The stem extenders are an integral part of the preoperative planning for total knee arthroplasty in patients with RA. It is also interesting to note that the authors have not used any such extenders in these patients.

The design of the prosthesis with less removal of the bone from the intercondylar notch (which is also our experience), appears promising and authors describe that this would make the revision easier. With 75.7% of patients in their follow-up being able to squat and sit cross legged, we foresee many patients would require revision due to polywear due to increased contact stresses with the polyethylene. But having said that, are the authors planning to alter the design to include options of stem extenders to make revision possible with Indus knee or they recommend the readers, prosthesis? The authors have not described the type of prosthesis used for the revision case in their series.

Can patients with high BMI, where fat thigh and the calf restrict the high flexion in the post operative periods, can get the benefits of this design? Can the preoperative deformity, range of motion, quadriceps strength, mobility status and obesity be confounding factors in post operative outcome in this population?
